# Living arrangement modifies the associations of loneliness with adverse health outcomes in older adults: evidence from the CLHLS

**DOI:** 10.1186/s12877-021-02742-5

**Published:** 2022-01-17

**Authors:** Kai Wei, Yong Liu, Junjie Yang, Nannan Gu, Xinyi Cao, Xudong Zhao, Lijuan Jiang, Chunbo Li

**Affiliations:** 1 Shanghai Key Laboratory of Psychotic Disorders, Shanghai Mental Health Center, Shanghai Jiao Tong University School of Medicine, 600 South Wanping Road, Shanghai, 200030 People’s Republic of China; 2 Institute of Psychology and Behavioral Science, Shanghai Jiao Tong University, Shanghai, 200030 People’s Republic of China; 3 CAS Center for Excellence in Brain Science and Intelligence Technology (CEBSIT), Chinese Academy of Science, Shanghai, People’s Republic of China; 4 Brain Science and Technology Research Center, Shanghai Jiao Tong University, Shanghai, People’s Republic of China

**Keywords:** Living arrangements, Loneliness, Health outcomes, Cohort study, Effect modificaiton

## Abstract

**Background:**

Although it has been suggested that loneliness is a risk factor for adverse health outcomes, living arrangement may confound the association. This study aimed to investigate whether the associations of loneliness with adverse health outcomes differ in community-dwelling older adults according to different living arrangements.

**Methods:**

In the 2008/2009 wave of Chinese Longitudinal Healthy Longevity Survey, 13,738 community-dwelling older adults (≥65 years) were included for analyses. Living arrangements and loneliness were assessed. Health outcomes including cognitive and physical functions were assessed using MMSE, ADL/IADL scales and Frailty Index in the 2008/2009 and 2011/2012 waves; mortality was assessed in the 3-year follow-up from 2008/2009 to 2011/2012. The effect modificaitons of loneliness on adverse health outcomes by living arrangements were estimated using logistic regression or Cox proportional hazards regression models.

**Results:**

Living alone older adults were significantly more likely to be lonely at baseline (52% vs 29.5%, OR = 1.90, 95% CI = 1.67–2.16, *P* < 0.001), compared with those living with others. Loneliness in older adults was a significant risk factor for prevalent cognitive impairment and frailty, and 3-year mortality, especially among those who lived with others (OR = 1.32, 95% CI = 1.15–1.52, *P* < 0.001; OR = 1.39, 95% CI = 1.24–1.57, *P* < 0.001; HR = 1.14, 95% CI = 1.05–1.24, *P* = 0.002, respectively). In contrast, among the living alone older adults, loneliness was only significantly associated with higher prevalence of frailty (OR = 1.42, 95% CI = 1.07–1.90, *P* = 0.017). Living arrangement significantly modified the associations of loneliness with prevalent cognitive impairment and 3-year mortality (*P* values for interaction = 0.005 and 0.026, respectively).

**Conclusions:**

Living arrangement modifies the associations of loneliness with adverse health outcomes in community-dwelling older adults, and those who lived with others but felt lonely had worse cognitive and physical functions as well as higher mortality. Special attention should be paid to this population and more social services should be developed to reduce adverse health outcomes, in order to improve their quality of life and promote successful aging.

**Supplementary Information:**

The online version contains supplementary material available at 10.1186/s12877-021-02742-5.

## Background

Older adults encounter transitions such as physical ageing, diminished resilience, decreased social relationships and loss of intimate relationships, which may reduce social connectedness or ability to participate in social network activities, and make them more susceptible to be lonely [1–3]. Loneliness can be explained as the lack of “meaningful” social relationships [1] or the discrepancy between one’s desired relationships and one’s actual relationships, either in quantity or quality [4]. It is conceptually tied to the magnitude of one’s social network, but mainly depends on how that individual subjectively perceives those relationships and how satisfied he/she is with the types of support received from those relationships, thus may also be unrelated to objective social conditions [3, 5]. Loneliness is a major source of suffering among older adults [6]. It has been found that loneliness increased the risk of developing dementia among older adults especially in men [7], and was associated with mental disorders such as depression, physical decline and increased risk of death [4, 8, 9].

As filial piety of the Confucius culture prevails in China, co-residence is valued as the most desirable living arrangement for older adults in community [10]. However, with the development of our society and increasing preferences for individual privacy and independence, older adults’ desire for living alone is changing. Some older adults may choose to live alone as they have better socioeconomic status and prefer a lifestyle of freedom and privacy [11], while others may be childless widows and do not have anyone to live with [12]. Living alone is often accompanied by a decreased level of family/social support and health care utilization, leading to social isolation and life challenges for older adults [13, 14]. Studies have found inconsistent associations of living alone with adverse health outcomes. Some found that living alone was a risk factor for cognitive impairment and mortality [15, 16], and older adults living with others had better ﻿psychological well-being [17]; while other studies found that living alone older adults had fewer physical disabilities and lower mortality risk [12].

Although living alone older adults are more likely to be lonely compared with their counterparts, and there is considerable overlap between living alone and loneliness, they are distinct definitions [3, 12]: living alone is an objective measure of one’s living arrangements, while loneliness is a subjective emotional experience of one’s personal relationships [18]. To date, few studies have investigated whether the impacts of loneliness on adverse health outcomes in community-dwelling older adults differ by living arrangements, and it remains unclear the extent to which loneliness affects health in older adults who live alone or otherwise. Therefore, our study aimed to assess the associations of loneliness with cognitive and physical functions and mortality, and to determine whether living arrangement modifies these associations among community-dwelling older adults aged 65 years or above in China.

## Methods

### Study design and participants

The Chinese Longitudinal Healthy Longevity Survey (CLHLS) is an ongoing, prospective cohort study of community-dwelling Chinese older adults [19, 20]. It covers the majority of the provinces in China and aims to investigate the factors associated with healthy longevity of Chinese. Started in 1998, the follow-ups have been conducted every 2 to 3 years. To reduce attrition, new participants are continually enrolled as death and lost-to-follow-up are inevitable. Trained interviewers with a structured questionnaire conduct the survey from door to door. Written informed consent was obtained from all participants and/or their proxy respondents, and the study was approved by the Research Ethics Committee of Peking University (IRB00001052–13074). A weight of age-sex-residence in the sample with the distribution of the total population was employed to reflect the unique sampling design [21]. All methods were performed in accordance with the relevant guidelines and regulations.

Our study utilized data from the 2008/2009 wave (baseline), which recruited the highest number of participants across all waves. 16,954 older adults in total were initially interviewed. We excluded 391 participants younger than 65 years, 308 participants living in an institution [as they were much older (93.1 ± 9.1 years), and institution living was different from community-dwelling], 2383 participants without definite status of loneliness [who were also much older (96.6 ± 7.7 years) and mostly cognitively impaired (96%)] and 134 demented older adults. Our final cross-sectional analyses included 13,738 community-dwelling older adults, among whom 54.8% (7524/13738) survived, 29.4% (4041/13738) died, and 15.8% (2173/13738) were lost in the 3-year follow-up (the 2011/2012 wave, see Table S1 for detailed information, the flow chart was shown in Fig. S1). Generally, the sociodemographic factors, socioeconomic status, and physical and cognitive functions of older adults who were lost in follow-up fell in between those who survived and died.

### Measurements

We used the data of living arrangements and loneliness at baseline, and assessed the associations of loneliness with adverse health outcomes in the total sample and stratified by living arrangements. Adverse health outcomes included cognitive impairment, functional limitation and frailty at both baseline and the 3-year follow-up, and 3-year mortality from 2008/2009 to 2011/2012.

#### Assessment of living arrangements and loneliness

Living arrangements were assessed using the question “Who do you live with?” with responses including ‘living with family (including house maid)’ and ‘living alone (LA)’. The former was defined as “not living alone/living with others (NLA, 83.1%)”.

Loneliness was assessed via the question “Do you feel lonely or isolated?” with answers ‘always’, ‘often’, ‘sometimes’, ‘seldom’, and ‘never’, which has been demonstrated to be feasible for loneliness assessment by previous studies [22, 23]. For the purpose of statistical analysis, we recoded the responses into a dichotomous variable: ‘always’, ‘often’, and ‘sometimes’ were defined as “feeling lonely/with loneliness (FL, 33.3%)”, ‘seldom’ and ‘never’ as “not feeling lonely/without loneliness (NFL, 66.7%)”.

#### Adverse health outcomes

##### Cognitive impairment

The CLHLS used the Chinese version of the Mini-mental State Examination (MMSE), validity and reliability of which have been verified [19, 20], as a measure of cognitive function at each wave. The total scores range from 0 to 30, with higher scores representing better cognitive function. We used education-adjusted criteria to define “cognitive impairment”: for participants without formal education, MMSE score ≤ 17 was defined as CI; for those with 1–6-year education, MMSE score ≤ 20 was defined as CI; for those with more than 6-year education, MMSE score ≤ 24 was defined as CI [24, 25].

##### Functional limitation

The Katz Basic Activities of Daily Living (ADL) Scale and Lawton Instrumental Activities of Daily Living (IADL) Scale were used to assess participants’ physical function. Having difficulty in performing any one or more of the ADL tasks (6 items: bathing, dressing, toileting, transfers, continence and eating) was defined as having ADL limitation; having difficulty in performing any one or more of the IADL tasks (8 items: be able to go outside to visit neighbors, shop by oneself, make food by oneself, wash clothes by oneself, walk one kilometer, carry 5 kg weight, crouch and stand 3 times, take public transportion) was defined as having IADL limitation. Participants with either ADL or IADL limitation were defined as functional limitation.

##### Frailty assessment

Our Frailty Index (FI) was the same as the previous CLHLS studies [26, 27]. FI included 39 self-reported items, including functional limitations, cognitive function, self-reported health status, interviewer-rated health status, mental health, auditory and visual ability, heart rhythm, and chronic diseases. We scored each term as 0 (absence of deficit) or 1 (presence of deficit) for 38 of 39 terms, and scored 1 term as 2 if the participants reported 2 or more serious illnesses that caused hospitalization or being bedridden in the past 2 years. FI score was equal to the number of reported deficits divided by the total number of included deficits. It was a continuous variable ranging from 0 to 1, with a higher value indicating severer frailty. The continuous FI score was classified into non-frailty (FI ≤ 0.21) and frailty (FI > 0.21) following a previous report [26, 27].

##### Mortality

Mortality was measured by survival status and duration of exposure to death. The survival status was measured by whether a respondent interviewed in the 2008/2009 wave died or survived at the 2011/2012 wave. The exposure duration for a survivor was measured by number of months between the interview date in the 2008/2009 and 2011/2012 waves. For those who died before the 2011/2012 wave, the exposure time was measured by the time interval between date of death and the interview date in the 2008/2009 wave. The date of death was collected from officially issued death certificates whenever available, otherwise the next-of-kin and local residential committees were consulted. The average follow-up period of all participants was 3.4 (±1.5) years, with 1.5 (±0.9) years for deceased participants, and 4.4 (±0.2) years for survived participants. The data quality of mortality in the CLHLS has been proved to be high [26].

#### Covariates

Measures of sociodemographic characteristics at baseline included age, gender, race (Han-Chinese or minority), marital status [married or single/separated/divorced/widowed (SDW)], residence (rural or city/town), occupation (non-professional or professional), education (< 1 year or ≥ 1 year), BMI, smoking (never/past or current), alcohol drinking (never/past or current). Socioeconomic status included sufficient financial support (yes or no), economic independence (yes or no), adequate medical service (yes or no), and public medical payment (yes or no). Dietary habits were assessed via the questions “How often do you eat fruit/vegetable, and drink tea?” with answers ‘everyday’, ‘almost everyday’ and ‘often’ defined as “eating fruits/eating vegetables/drinking tea”. Living preference was assessed via the question “What kind of living arrangement do you like best?”, with answers ‘living alone (or only with spouse) regardless of proximity to children’, ‘living alone (or only with spouse) with close proximity to children’, ‘live with children’, ‘institution’ and ‘don’t know’. The former two answers were combined as “preferring LA”, and the rest as “preferring NLA”, “preferring institution” and “NA”. Social/leisure activity score was calculated in the way same as a previous study [24], with a high score representing a high frequency of social and leisure activities. Physical exercise was assessed via the question “Do you perform physical exercise or not at present?” with answers ‘yes’ or ‘no’. Self-reported health was assessed via the question “How do you rate your health status?” with answers ‘bad’ and ‘very bad’ defined as “poor self-reported health”. Interviewer-rated health was assessed by interviewers, with ‘moderately ill’ and ‘very ill’ defined as “poor interviewer-rated health”. Comorbidity was assessed via whether suffering from 24 common chronic diseases including hypertension, diabetes, heart disease and stroke. Serious illness in the past 2 years was defined as “illness that causes hospitalization or being bedridden all the year around”. Hearing and visual ability were also assessed.

### Statistical analysis

Categorical variables were presented as numbers (percentages), and continuous variables were presented as means (SD). Differences in the distribution of categorical variables among groups were tested by χ^2^ test. For continuous variables, the F test or Kruskall-Wallis test was used for comparison between different groups. Logistic regression models were performed to assess the association of living alone with loneliness, as well as the associations of loneliness with cognitive impairment, functional limitation and frailty in the total sample and stratified by living arrangements, and calculate the corresponding odds ratios (ORs) and 95% confidence intervals (CIs). Cox proportional hazards regression model was performed to assess the associations of loneliness with 3-year mortality in the total sample and stratified by living arrangements, and calculate the hazard ratios (HRs) and 95% CI. The above regression models were also performed within strata of age groups (< 80 or ≥ 80 years) and genders. To test whether living arrangement was an effect modifier, interaction terms between living arrangements and loneliness for the prevalent adverse health outcomes were assessed in logistic regression models, adjusted for baseline values of age, gender, race, marital status, residence, occupation, education, BMI, smoking, alcohol drinking, living preferences, socioeconomic status, dietary habits, social/leisure activity score, physical exercise, poor self-rated health, poor interviewer-rated health, comorbidities (≥2), hypertension, diabetes, heart disease, stroke, serious illness in the past 2 years, hearing problem, visual impairment, cognitive impairment, functional limitation, and frailty. OR estimates for prevalent cognitive impairment, functional limitation and frailty, and HR for 3-year mortality were adjusted for the same set of confounders cited above. As many variables changed from 2008/2009 to 2011/2012, interaction terms and OR estimates for incident cognitive impairment, functional limitation and frailty were adjusted for age, gender, race, education, occupation, hypertension, diabetes, heart disease, stroke, and changes of other variables from 2008/2009 to 2011/2012. The multicollinearity of the covariates adjusted in the above regression models was assessed by calculating their variance inflation factor (VIF) values (< 10 indicating no collinearity). In the longitudinal analyses, 9166 older adults who were not lonely at baseline were included for analyzing incident loneliness; 11,118 older adults without cognitive impairment at baseline were included for analyzing incident cognitive impairment; 7560 older adults without functional limitation at baseline were included for analyzing incident functional limitation; and 10,524 older adults without frailty at baseline were included for analyzing incident frailty. The acceptable level of significance was set as two-sided *P* < 0.05. Stata version 14.0 (StataCorp LP, College Station, TX, USA) was used for data analysis.

## Results

### Baseline characteristics by living arrangements and loneliness

As shown in Table 1, some of the factors associated with LA and FL were similar. In general, both LA and FL were more prevalent among older adults who were female, SDW, lived in rural, had non-professional occupations, less education, lower BMI, worse financial status, lower social/leisure activity score, and poor self-reported health. Meanwhile, fewer of the LA and FL older adults ate fruits and vegetables.

**Table 1:** Baseline Characteristics by Living Arrangements and Feelings of Loneliness

Characteristics	Total Sample (***N*** = 13,738)	NLA	LA	***P***	NFL	FL	***P***
		11,414 (83.1)	2324 (16.9)		9166 (66.7)	4572 (33.3)	
**Sociodemographic**							
Age (years)	85.7 (11.2)	85.7 (11.4)	85.8 (9.6)	0.968	84.6 (11.4)	88.0 (10.4)	**< 0.001**
Gender (female)	7550 (55.0)	6183 (54.2)	1367 (58.8)	**< 0.001**	4772 (52.1)	2778 (60.8)	**< 0.001**
Race (minority)	890 (6.5)	763 (6.7)	127 (5.5)	**0.029**	537 (5.9)	353 (7.7)	**< 0.001**
Marital status (SDW)	9101 (66.3)	6801 (59.6)	2300 (99.0)	**< 0.001**	5312 (58.0)	3789 (82.9)	**< 0.001**
Residence (rural)	8250 (60.1)	6769 (59.3)	1481 (63.7)	**< 0.001**	5265 (57.4)	2985 (65.3)	**< 0.001**
Occupation (professional)	1027 (7.5)	899 (7.9)	128 (5.5)	**< 0.001**	804 (8.8)	223 (4.9)	**< 0.001**
Education (≥1 year)	5489 (40.0)	4692 (41.2)	797 (34.4)	**< 0.001**	4024 (44.0)	1465 (32.1)	**< 0.001**
BMI (kg/m^2^)	20.4 (3.5)	20.5 (3.5)	20.0 (3.4)	**< 0.001**	20.7 (3.6)	19.8 (3.4)	**< 0.001**
Current smoker	2511 (18.3)	2075 (18.2)	436 (18.8)	0.509	1824 (19.9)	687 (15.0)	**< 0.001**
Current alcohol drinker	2457 (17.9)	2062 (18.1)	395 (17.0)	0.220	1793 (19.6)	664 (14.5)	**< 0.001**
Preferring LA	5583 (40.6)	3944 (34.6)	1639 (70.5)	**< 0.001**	3986 (43.5)	1597 (34.9)	**< 0.001**
**Socioeconomic status**							
Sufficient financial support	10,735 (78.1)	9048 (79.3)	1687 (72.6)	**< 0.001**	7608 (83.0)	3127 (68.4)	**< 0.001**
Economic independence	3769 (27.4)	3229 (28.3)	540 (23.2)	**< 0.001**	2898 (31.6)	871 (19.1)	**< 0.001**
Adequate medical service	12,720 (92.6)	10,699 (93.7)	2021 (87.0)	**< 0.001**	8780 (95.8)	3940 (86.2)	**< 0.001**
Public medical payment	1859 (13.5)	1597 (14.0)	262 (11.3)	**< 0.001**	1377 (15.0)	482 (10.5)	**< 0.001**
**Dietary habits**							
Fruit eating	5403 (39.3)	4703 (41.2)	700 (30.1)	**< 0.001**	3985 (43.5)	1418 (31.0)	**< 0.001**
Vegetable eating	12,183 (88.7)	10,221 (89.6)	1962 (84.4)	**< 0.001**	8246 (90.0)	3937 (86.1)	**< 0.001**
Tea drinking	5668 (41.3)	4743 (41.6)	925 (39.8)	0.115	3889 (42.5)	1779 (38.9)	**< 0.001**
**Physical health status**							
Social/leisure activity score (point)	3.5 (3.1)	3.6 (3.2)	3.0 (2.8)	**< 0.001**	3.9 (3.2)	2.8 (2.9)	**< 0.001**
Physical exercise	4115 (30.0)	3409 (29.9)	706 (30.4)	0.625	3055 (33.3)	1060 (23.2)	**< 0.001**
Poor self-reported health	2183 (15.9)	1759 (15.4)	424 (18.3)	**0.001**	1093 (11.9)	1090 (23.9)	**< 0.001**
Poor interviewer-rated health	1906 (13.9)	1620 (14.2)	286 (12.3)	**0.016**	926 (10.1)	980 (21.4)	**< 0.001**
Comorbidities (≥ 2)	6298 (45.9)	5281 (46.3)	1017 (43.8)	**0.027**	4161 (45.4)	2137 (46.8)	0.134
Hypertension	2790 (20.8)	2307 (20.7)	483 (21.3)	0.512	1864 (20.7)	926 (20.9)	0.853
Diabetes	377 (2.8)	323 (2.9)	54 (2.4)	0.171	256 (2.8)	121 (2.7)	0.700
Heart disease	1248 (9.3)	1074 (9.6)	174 (7.6)	**0.003**	864 (9.6)	384 (8.6)	0.071
Stroke	721 (5.3)	631 (5.6)	90 (3.9)	**0.001**	481 (5.3)	240 (5.4)	0.922
Serious illness in the past 2 years	2255 (16.4)	1939 (17.0)	316 (13.6)	**< 0.001**	1439 (15.7)	816 (17.9)	**0.001**
Hearing problem	2234 (16.3)	1938 (17.0)	296 (12.7)	**< 0.001**	1245 (13.6)	989 (21.6)	**< 0.001**
Visual impairment	2193 (16.0)	1832 (16.1)	361 (15.6)	0.535	1228 (13.4)	965 (21.2)	**< 0.001**
**Adverse health outcomes**							
Cognitive impairment	2590 (18.9)	2219 (19.5)	371 (16.0)	**< 0.001**	1405 (15.4)	1185 (26.0)	**< 0.001**
Functional limitation	6178 (45.0)	5286 (46.3)	892 (38.4)	**< 0.001**	3670 (40.0)	2508 (54.9)	**< 0.001**
Frailty	3214 (23.4)	2848 (25.0)	366 (15.8)	**< 0.001**	1770 (19.3)	1444 (31.6)	**< 0.001**

*Note.* Data presented as n (%) or mean (SD). NLA, not living alone; LA, living alone; NFL, not feeling lonely; FL, feeling lonely; SDW, Single/Separated/Divorced/Widowed

Compared with those who were NLA, LA older adults were more likely to be Han-Chinese and prefer LA, fewer had poor interviewer-rated health, ≥2 comorbidities (including heart disease and stroke), serious illness in the past 2 years, hearing problem, cognitive impairment, functional limitation and frailty. Compared with those who were NFL, FL older adults tended to be older and non-Han-Chinese, fewer were currently smoking, drinking alcohol and tea, or performing physical exercise, and more preferred NLA, had poor interviewer-rated health, serious illness in the past 2 years, hearing problem, visual impairment, cognitive impairment, functional limitation and frailty.

### Associations between living arrangements and loneliness

As shown in Table 2, compared with NLA, LA was significantly associated with higher rate of loneliness at baseline when no covariate, only sociodemographic characteristics and all confounders were adjusted (OR = 2.61, 95% CI = 2.38–2.85, *P* < 0.001; OR = 1.96, 95% CI = 1.75–2.19, *P* < 0.001; OR = 1. 90, 95% CI = 1.67–2.16, *P* < 0.001, respectively). At the 3-year follow-up, LA was significantly associated with higher risk of loneliness only when no covariate was adjusted (OR = 1.73, 95% CI = 1.46–2.05, *P* < 0.001); when sociodemographic characteristics were adjusted, LA was marginally associated with higher risk of loneliness (OR = 1.27, 95% CI = 1.00–1.62, *P* = 0.051); and LA was only associated with increasing trend in the risk of loneliness when all the confounders were adjusted (OR = 1.09, 95% CI = 0.84–1.43, *P* = 0.518).

**Table 2:** Associations of Living Alone with Prevalent and Incident Loneliness

	Loneliness						
	No. (%)	Model 1		Model 2		Model 3	
		OR (95% CI)	***P***	OR (95% CI)	***P***	OR (95% CI)	***P***
**Cross-sectional Analyses** ^**a**^							
NLA	3361 (29.5)	1.00		1.00		1.00	
LA	1211 (52.1)	2.61 (2.38–2.85)	**< 0.001**	1.96 (1.75–2.19)	**< 0.001**	1.90 (1.67–2.16)	**< 0.001**
**Longitudinal Analyses** ^**b**^							
NLA	1052 (22.3)	1.00		1.00		1.00	
LA	217 (34.0)	1.73 (1.46–2.05)	**< 0.001**	1.27 (1.00–1.62)	0.051	1.09 (0.84–1.43)	0.518

*Note.* NLA, not living alone; LA, living alone

^a^ Model 1: no covariate was adjusted. Model 2: Adjusted for age, gender, race, marital status, residence, occupation, education, BMI, smoking, alcohol drinking, and living preference. Model 3: Adjusted for the covariates in Model 2 and socioeconomic status, dietary habits, social/leisure activity score, physical exercise, poor self-rated health, poor interviewer-rated health, comorbidities (≥2), hypertension, diabetes, heart disease, stroke, serious illness in the past 2 years, hearing problem, visual impairment

^b^ For 9166 participants being not lonely at baseline. Model 1: no covariate was adjusted. Model 2: Adjusted for age, gender, race, occupation, education, and changes in marital status, residence, BMI, smoking, alcohol drinking and living preference. Model 3: Adjusted for the covariates in Model 2 and hypertension, diabetes, heart disease, stroke, and changes in socioeconomic status, dietary habits, social/leisure activity score, physical exercise, self-rated health, interviewer-rated health, comorbidity number, serious illness in the past 2 years, hearing problem and visual impairment from 2008/2009 to 2011/2012

### Effect modifications of loneliness on adverse health outcomes by living arrangements

As shown in Table 3, in cross-sectional analysis, adjusted for confounders, loneliness was a risk factor for cognitive impairment in the total sample (OR = 1.22, 95% CI = 1.07–1.38, *P* = 0.003). When stratified by living arrangements, compared with the NFL older adults, loneliness was not associated with cognitive impairment in the LA older adults (OR = 0.80, 95% CI = 0.58–1.09, *P* = 0.156), but showed higher OR in the NLA older adults (OR = 1.32, 95% CI = 1.15–1.52, *P* < 0.001) than in the total sample or in the LA ones. Living arrangement significantly modified the association of loneliness with prevalent cognitive impairment (*P* value for interaction =0.005). In the longitudinal analyses, after adjusted for confounders including changes of some variables from 2008/2009 to 2011/2012, living arrangement had no significant effect modification of loneliness on incident cognitive impairment (*P* value for interaction =0.607), although the same trend as cross-sectional analyses was shown. Living arrangement also had no effect modification of loneliness on both functional limitation and frailty at baseline and follow-up (*P* values for interaction =0.446–0.989, Table 4 and 5). At baseline, FL older adults were significantly more likely to be frail compared with the NFL ones, no matter in the total sample or stratified by living arrangements (total sample: OR = 1.40, 95% CI = 1.25–1.56, *P* < 0.001; for the NLA older adults: OR = 1.39, 95% CI = 1.24–1.57, *P* < 0.001; for the LA older adults: OR = 1.42, 95% CI = 1.07–1.90, *P* = 0.017, Table 5). As shown in Table 6, compared with the NFL older adults, loneliness was significantly associated with higher rate of 3-year mortality in the total sample (HR = 1.10, 95% CI = 1.02–1.19, *P* = 0.012); this association remained significant only in the NLA older adults (HR = 1.14, 95% CI = 1.05–1.24, *P* = 0.002) but not the LA ones (HR = 0.92, 95% CI = 0.76–1.11, *P* = 0.403). Living arrangement significantly modified the association of loneliness with 3-year mortality (*P* value for interaction =0.026).

**Table 3:** Modification of the Effect of Loneliness on Cognitive Impairment by Living Arrangement

	No Loneliness		Loneliness		Odds Ratio (95% CI) for the Association Between Loneliness and Cognitive Impairment Within Each Stratum of Living Arrangement
	N With/Without Cognitive Impairment	Odds Ratio (95% CI)	N With/Without Cognitive Impairment	Odds Ratio (95% CI)	
**Cross-sectional Analyses** ^**a**^					
Total Sample ^c^	1405/7745	1.0 (reference)	1185/3373	1.22 (1.07–1.38) *P* = 0.003	–
NLA	1241/6799	1.0 (reference)	978/2372	1.32 (1.15–1.52) *P* < 0.001	1.32 (1.15–1.52) *P* < 0.001
LA	164/946	1.13 (0.87–1.47) *P* = 0.348	207/1001	0.94 (0.73–1.20) *P* = 0.604	0.80 (0.58–1.09) *P* = 0.156
**Longitudinal Analyses** ^b^					
Total Sample ^c^	776/4088	1.0 (reference)	394/1371	1.01 (0.81–1.27) *P* = 0.926	–
NLA	675/3626	1.0 (reference)	290/910	1.04 (0.81–1.34) *P* = 0.748	1.04 (0.80–1.34) *P* = 0.783
LA	101/462	1.04 (0.69–1.57) *P* = 0.844	104/461	0.94 (0.63–1.43) *P* = 0.787	0.91 (0.52–1.61) *P* = 0.757

*Note.* NLA, not living alone; LA, living alone

^a^ Measure of effect modification on multiplicative scale: OR (95% CI) = 0.63 (0.45–0.87), *P* = 0.005. Adjusted for age, gender, race, marital status, residence, occupation, BMI, smoking, alcohol drinking, living preference, socioeconomic status, dietary habits, social/leisure activity score, physical exercise, poor self-rated health, poor interviewer-rated health, comorbidities (≥2), hypertension, diabetes, heart disease, stroke, serious illness in the past 2 years, hearing problem, visual impairment, functional limitation, and frailty. Education was not adjusted as we used education-adjusted criteria to define “cognitive impairment”

^b^ Measure of effect modification on multiplicative scale: OR (95% CI) = 0.87 (0.51–1.48), *P* = 0.607. For 11,118 participants without cognitive impairment at baseline. Adjusted for age, gender, race, occupation, hypertension, diabetes, heart disease, stroke, and changes in marital status, residence, BMI, smoking, alcohol drinking, socioeconomic status, dietary habits, social/leisure activity score, physical exercise, self-rated health, interviewer-rated health, comorbidity number, serious illness in the past 2 years, hearing problem, visual impairment, functional limitation, and frailty from 2008/2009 to 2011/2012

^**c**^ Living arrangements were also adjusted

**Table 4:** Modification of the Effect of Loneliness on Functional Limitation by Living Arrangement

	No Loneliness		Loneliness		Odds Ratio (95% CI) for the Association Between Loneliness and Functional Limitation Within Each Stratum of Living Arrangement
	N With/Without Functional Limitation	Odds Ratio (95% CI)	N With/Without Functional Limitation	Odds Ratio (95% CI)	
**Cross-sectional Analyses** ^**a**^					
Total Sample ^c^	3670/5496	1.0 (reference)	2508/2064	1.06 (0.95–1.18) *P* = 0.305	–
NLA	3277/4776	1.0 (reference)	2009/1352	1.08 (0.96–1.22) *P* = 0.208	1.07 (0.95–1.21) *P* = 0.259
LA	393/720	0.62 (0.51–0.76) *P* < 0.001	499/712	0.61 (0.50–0.74) *P* < 0.001	1.00 (0.79–1.26) *P* = 0.987
**Longitudinal Analyses** ^b^					
Total Sample ^c^	1226/2585	1.0 (reference)	549/714	1.09 (0.90–1.32) *P* = 0.400	–
NLA	1066/2285	1.0 (reference)	359/468	1.08 (0.87–1.35) *P* = 0.485	1.07 (0.85–1.34) *P* = 0.558
LA	160/300	0.72 (0.52–0.99) *P* = 0.044	190/246	0.78 (0.56–1.10) *P* = 0.157	1.13 (0.75–1.69) *P* = 0.570

*Note.* NLA, not living alone; LA, living alone

^a^ Measure of effect modification on multiplicative scale: OR (95% CI) = 0.91 (0.70–1.17), *P* = 0.446. Adjusted for age, gender, race, marital status, residence, occupation, education, BMI, smoking, alcohol drinking, living preference, socioeconomic status, dietary habits, social/leisure activity score, physical exercise, poor self-rated health, poor interviewer-rated health, comorbidities (≥2), hypertension, diabetes, heart disease, stroke, serious illness in the past 2 years, hearing problem, visual impairment, and cognitive impairment. Frailty was not adjusted as functional limitation was included in the calculation of FI score

^b^ Measure of effect modification on multiplicative scale: OR (95% CI) = 1.01 (0.66–1.56), *P* = 0.951. For 7560 participants without functional limitation at baseline. Adjusted for age, gender, race, occupation, education, hypertension, diabetes, heart disease, stroke, and changes in marital status, residence, BMI, smoking, alcohol drinking, socioeconomic status, dietary habits, social/leisure activity score, physical exercise, self-rated health, interviewer-rated health, comorbidity number, serious illness in the past 2 years, hearing problem, visual impairment, and cognitive impairment from 2008/2009 to 2011/2012

^**c**^ Living arrangements were also adjusted

**Table 5:** Modification of the Effect of Loneliness on Frailty by Living Arrangement

	No Loneliness		Loneliness		Odds Ratio (95% CI) for the Association Between Loneliness and Frailty Within Each Stratum of Living Arrangement
	N With/Without Frailty	Odds Ratio (95% CI)	N With/Without Frailty	Odds Ratio (95% CI)	
**Cross-sectional Analyses** ^**a**^					
Total Sample ^c^	1770/7396	1.0 (reference)	1444/3128	1.40 (1.25–1.56) *P* < 0.001	–
NLA	1633/6420	1.0 (reference)	1215/2146	1.39 (1.24–1.57) *P* < 0.001	1.39 (1.24–1.57) *P* < 0.001
LA	137/976	0.47 (0.37–0.60) *P* < 0.001	229/982	0.66 (0.53–0.81) *P* < 0.001	1.42 (1.07–1.90) *P* = 0.017
**Longitudinal Analyses** ^b^					
Total Sample ^c^	1110/3730	1.0 (reference)	493/1241	1.20 (0.99–1.46) *P* = 0.067	–
NLA	970/3282	1.0 (reference)	346/816	1.21 (0.97–1.51) *P* = 0.090	1.20 (0.96–1.50) *P* = 0.103
LA	140/448	0.85 (0.60–1.21) *P* = 0.373	147/425	0.99 (0.70–1.41) *P* = 0.969	1.08 (0.73–1.60) *P* = 0.715

*Note.* NLA, not living alone; LA, living alone

^a^ Measure of effect modification on multiplicative scale: OR (95% CI) = 1.00 (0.74–1.36), *P* = 0.989. Adjusted for age, gender, race, marital status, residence, occupation, education, BMI, smoking, alcohol drinking, living preference, socioeconomic status, dietary habits, social/leisure activity score, physical exercise, comorbidities (≥2). Poor self-rated health, poor interviewer-rated health, hypertension, diabetes, heart disease, stroke, serious illness in the past 2 years, hearing problem, visual impairment, cognitive impairment, and functional limitation were not adjusted as they were included in the calculation of FI score

^b^ Measure of effect modification on multiplicative scale: OR (95% CI) = 0.96 (0.61–1.51), *P* = 0.863. For 10,524 participants without frailty at baseline. Adjusted for age, gender, race, occupation, education, and changes in marital status, residence, BMI, smoking, alcohol drinking, socioeconomic status, dietary habits, social/leisure activity score, physical exercise, and comorbidity number from 2008/2009 to 2011/2012

^**c**^ Living arrangements were also adjusted

**Table 6:** Modification of the Effect of Loneliness on 3-year Mortality by Living Arrangement

	No Loneliness		Loneliness		Hazard Ratio (95% CI) for the Association Between Loneliness and 3-year Mortality Within Each Stratum of Living Arrangement
	3-year Mortality Per 100 Person-years	Hazard Ratio (95% CI)	3-year Mortality Per 100 Person-years	Hazard Ratio (95% CI)	
**Longitudinal Analyses**					
Total Sample ^**a**^	8.6	1.0 (reference)	13.4	1.10 (1.02–1.19) *P* = 0.012	–
NLA	8.6	1.0 (reference)	14.8	1.14 (1.05–1.24) *P* = 0.002	1.14 (1.05–1.24) *P* = 0.002
LA	8.5	1.07 (0.91–1.25) *P* = 0.399	10.0	1.01 (0.87–1.17) *P* = 0.918	0.92 (0.76–1.11) *P* = 0.403

*Note.* NLA, not living alone; LA, living alone

Measure of effect modification on multiplicative scale: OR (95% CI) = 0.74 (0.57–0.96), *P* = 0.026. Adjusted for age, gender, race, marital status, residence, occupation, education, BMI, smoking, alcohol drinking, living preference, socioeconomic status, dietary habits, social/leisure activity score, physical exercise, poor self-rated health, poor interviewer-rated health, comorbidities (≥2), hypertension, diabetes, heart disease, stroke, serious illness in the past 2 years, hearing problem, visual impairment, cognitive impairment, functional limitation, and frailty

^a^ Living arrangements were also adjusted

Age and gender also affected the associations of loneliness with adverse health outcomes in the total sample or stratified by living arrangements. As shown in Table S2, loneliness was associated with higher prevalence of cognitive impairment especially in older adults ≥80 years and older females (OR = 1.24, *P* = 0.002; OR = 1.21, *P* = 0.022, respectively); when stratified by living arrangements, the associations remained significant only in the NLA older adults (NLA older adults ≥80 years: OR = 1.34, *P* < 0.001; NLA older males: OR = 1.36, *P* = 0.012; NLA older females: OR = 1.32, *P* = 0.002) but not the LA ones. Loneliness was also associated with incident cognitive impairment at follow-up in the NLA older males (OR = 1.59, *P* = 0.036). The significant effect modifications of loneliness on prevalent cognitive impairment by living arrangement were found in older adults ≥80 years and older females (*P* values for interaction =0.006 and 0.018, respectively). Living arrangement had no effect modification of loneliness on both functional limitation and frailty at baseline and follow-up, within strata of age groups and genders (*P* values for interaction =0.099–0.993, Table S3-S4). For frailty, loneliness was associated with higher prevalence of frailty in both age groups and genders (ORs = 1.36–1.87, all *P* < 0.001), especially in older adults < 80 years and older males (ORs = 1.87 and 1.46); when stratified by living arrangements, all the above associations remained significant in the NLA ones (ORs = 1.33–1.87); in the LA older adults, loneliness was significantly associated with prevalent frailty only in older females (OR = 1.66, *P* = 0.006). Meanwhile, loneliness was also significantly associated with incident frailty at follow-up in older females of the total sample (OR = 1.29, *P* = 0.042, Table S4). Loneliness was a significant risk factor for 3-year mortality especially in older adults < 80 years and older females (HR = 1.50, *P* = 0.002; HR = 1.13, *P* = 0.015, respectively); when stratified by living arrangements, the associations were significant in both age groups and females only in the NLA older adults (HRs = 1.11–1.55, *P* = 0.002–0.016, Table S5). The significant effect modification of loneliness on 3-year mortality by living arrangement was only found in older adults ≥80 years (*P* value for interaction =0.025), and marginally significant in older females (*P* value for interaction =0.070).

## Discussion

Living alone and loneliness are related but distinct concepts. Our study found that community-dwelling LA or FL older adults shared some common characteristics, but also had their respective related factors. LA older adults were more likely to be Han-Chinese and prefer LA, as well as had better physical and cognitive functions; while FL older adults were on the contrary. Loneliness was more prevalent among older adults who lived alone (1211/2324, 52.1%) than those living with others (3361/11414, 29.5%) in our study, and the proportions were much higher than 24.9 and 5.6% in a French study [28], indicating that the situation of loneliness among Chinese older adults was more severe. LA was also associated with higher incidence of loneliness in the longitudinal analyses (LA: 34% vs NLA: 22.3%).

In our study, nearly all the LA older adults were SDW (99%) and widowed older adults accounted for 96% in those who were SDW, which meant widowhood was the major cause for them to live alone. The death of a spouse signifies the loss of a significant attachment figure that likely provided a meaningful and intimate source of social support, and may lead to loneliness [29]. SDW older adults may also choose to live with others, as 75% (6801/9101) of the SDW older adults did not live alone and 67% (6102/9101) of them preferred NLA. It is reasonable for us to consider that living alone was most likely a personal choice for some older adults, as nearly 71% (1639/2324) of the LA older adults preferred LA (much more than 41% in the total sample). However, majority of those who preferred LA [up to 71% (3944/5583)] lived with others and about 35% (3944/11414) of the NLA older adults preferred LA, demonstrating a disconnection between their preferences for living arrangements and actual living arrangements. On the other hand, 94% (7160/7658) of older adults who preferred NLA achieved their preferences.

Consistent with previous studies [4, 8, 23], after adjusting for confounders, loneliness was a risk factor for prevalent cognitive impairment and frailty, as well as 3-year mortality in the total sample. When stratified by living arrangements, we found that although the prevalence of loneliness was lower in the NLA older adults, lonely NLA older adults accounted for one-fifth of the total sample and encountered more severe adverse health outcomes, hence it is imperative to focus on the effects of loneliness in this subpopulation. Loneliness remains a significant risk factor for prevalent cognitive impairment and mortality in the NLA older adults but not the LA ones, although the associations of loneliness with prevalent frailty were significant in both the NLA and LA older adults. In addition to the modifying effect of living arrangement, age and gender could also modify the associations of loneliness with prevalent cognitive impairment and mortality: older adults ≥80 years, older males and older females had higher risk of prevalent cognitive impairment; older adults < 80 years and older females were especially more likely to die at the 3-year follow-up. These results were consistent with previous studies [7, 9, 23], but further demonstrated the role of age in modifying the associations of loneliness with cognitive impairment and mortality. Our findings concluded that when managing the impacts of loneliness on different adverse health outcomes, not only living arrangement but age and gender should be considered when formulating individualized strategies.

As filial piety seems to be achieved once older adults live with others (mainly their children), loneliness in this subpopulation is easily neglected. One Singapore study has found that older adults who were more likely to feel lonely were those who lived with their children and perceived they failed to fulfill their responsibilities [18]. The difference between perceived and expected amount of support they derived from their children led to the occurrence of loneliness [18]. Therefore, in community services, the range of loneliness screening should be expanded to find the “NLA but FL” older adults. Our study found that better socioeconomic status, higher social/leisure activity score and more physical exercise were generally associated with not only reduced loneliness, but also reduced adverse health outcomes (data not shown). Therefore, measures including improving older adults’ socioeconomic conditions, increasing their social/leisure activities and physical exercise, providing enough social support, more accurately understanding and satisfying their real needs should be adopted. Adult children play an irreplaceable role for managing loneliness in these older adults [30], thus individualized strategy should also be formulated based on the situation of each family to reduce loneliness so as to prevent adverse health outcomes.

An important contribution of this study is that we find living arrangement modifies the associations of loneliness with prevalent cognitive impairment and mortality, which has not been reported before. Loneliness has different impacts on older adults’ health according to different living arrangements, age and genders. Therefore, these factors need to be taken into consideration when formulating related policies at familial and societal levels. However, some limitations still exist in our study. First, loneliness was assessed via one single question but not a scale, which made it hard for us to evaluate other dimensions (e.g. the varying degrees) of loneliness and may cause some bias. Second, compared with the NLA older adults, those who lived alone had better physical and cognitive functions, which may influence the effect modifications of living arrangements on the associations of loneliness with adverse health outcomes. In our regression models, these factors were adjusted as covariates, which may guarantee the reliability of our results to some extent. Third, living arrangements and loneliness were both dynamic, and we only considered baseline living arrangements and loneliness, which may be the main reason for insignificant associations of loneliness with incident adverse health outcomes at follow-up. However, when analyzing the associations of loneliness with incident adverse health outcomes, changes of some covariates including sociodemographic, socioeconomic, dietary habits, and physical characteristics were adjusted in our logistic regression models, which may also guarantee the reliability of our results to some extent. In the future research, scales with good reliability and validity should be adopted to evaluate more dimensions of loneliness, and factors associated with dynamic changes of loneliness should be further investigated, so as to develop corresponding interventions to improve physical and cognitive functions and reduce mortality in community-dwelling older adults.

## Conclusions

Living arrangement modifies the associations of loneliness with adverse health outcomes in community-dwelling older adults, and those who lived with others but felt lonely had worse cognitive and physical functions as well as higher mortality. Special attention should be paid to this population and more social services should be developed to reduce adverse health outcomes, in order to improve their quality of life and promote successful aging.

## Supplementary Information

**Additional file 1.**

**Additional file 2.**

## Data Availability

The datasets used and analyzed during the current study are available from Peking University Open Research Data Platform. (https://opendata.pku.edu.cn/dataverse/CHADS?from=timeline&isappinstalled=0).

## Abbreviations

CLHLS: Chinese Longitudinal Healthy Longevity Study
NLA: not living alone
LA: living alone
NFL: not feeling lonely
FL: feeling lonely
SDW: Single/Separated/Divorced/Widowed
MMSE: Mini-mental State Examination
ADL: Activities of Daily Living
IADL: Instrumental Activities of Daily Living.
