# Supplementary material for: Living arrangement modifies the associations of loneliness with adverse health outcomes in older adults: evidence from the CLHLS
Source: BMC Geriatr. 2022 Jan 17;22:59. doi: 10.1186/s12877-021-02742-5 (PMC8764854; doi:10.1186/s12877-021-02742-5)
Supplement: Supplementary file 1 — Additional file 1. [file 12877_2021_2742_MOESM1_ESM.docx]

**Table S1. Baseline Characteristics by Status from 2008/2009 to 2011/2012**

| **Characteristics** | **Surviving**  **7524 (54.8)** | **Died**  **4041 (29.4)** | **Lost in Follow-up**  **2173 (15.8)** | ***P*** |
| --- | --- | --- | --- | --- |
| **Socio-demographic** |  |  |  |  |
| Age (years) | 81.9 (10.5) | 92.2 (8.9) | 86.9 (11.3) | **<0.001** |
| Gender (female) | 4030 (53.6) | 2294 (56.8) | 1226 (56.4) | **<0.001** |
| Race (minority) | 481 (6.4) | 334 (8.3) | 75 (3.5) | **<0.001** |
| Marital status (SDW) | 4270 (56.8) | 3274 (81.0) | 1557 (71.7) | **<0.001** |
| Residence (rural) | 4626 (61.5) | 2669 (66.1) | 955 (44.0) | **<0.001** |
| Occupation (professional) | 612 (8.2) | 195 (4.8) | 220 (10.1) | **<0.001** |
| Education (≥ 1 year) | 3361 (44.8) | 1219 (30.2) | 909 (42.0) | **<0.001** |
| BMI (kg/m^2^) | 20.8 (3.6) | 19.6 (3.4) | 20.5 (3.4) | **<0.001** |
| Current smoker | 1554 (20.7) | 640 (15.8) | 317 (14.6) | **<0.001** |
| Current alcohol drinker | 1510 (20.1) | 661 (16.4) | 286 (13.2) | **<0.001** |
| Prefer living alone | 3546 (47.1) | 1183 (29.3) | 854 (39.3) | **<0.001** |
| **Socioeconomic status** |  |  |  |  |
| Sufficient financial support | 5860 (77.9) | 3101 (76.7) | 1774 (81.6) | **<0.001** |
| Economic independence | 2455 (32.6) | 578 (14.3) | 736 (33.9) | **<0.001** |
| Adequate medical service | 7031 (93.5) | 3684 (91.2) | 2005 (92.3) | **<0.001** |
| Public medical payment | 962 (12.8) | 406 (10.1) | 491 (22.6) | **<0.001** |
| **Dietary habits** |  |  |  |  |
| Fruit eating | 3001 (39.9) | 1386 (34.3) | 1016 (46.8) | **<0.001** |
| Vegetable eating | 6818 (90.6) | 3449 (85.4) | 1916 (88.2) | **<0.001** |
| Tea drinking | 3230 (43.0) | 1486 (36.8) | 952 (43.8) | **<0.001** |
| **Physical health status** |  |  |  |  |
| Social/leisure activity score (point) | 4.2 (3.1) | 2.3 (2.6) | 3.5 (3.2) | **<0.001** |
| Physical exercise | 2573 (34.2) | 839 (20.8) | 703 (32.4) | **<0.001** |
| Poor self-reported health | 1069 (14.2) | 798 (19.8) | 316 (14.5) | **<0.001** |
| Poor interviewer-rated health | 720 (9.6) | 858 (21.2) | 328 (15.1) | **<0.001** |
| Comorbidities (≥ 2) | 3491 (46.4) | 1730 (42.8) | 1077 (49.6) | **<0.001** |
| Hypertension | 1641 (22.2) | 678 (17.2) | 471 (22.3) | **<0.001** |
| Diabetes | 216 (2.9) | 65 (1.7) | 96 (4.6) | **<0.001** |
| Heart disease | 699 (9.4) | 291 (7.4) | 258 (12.2) | **<0.001** |
| Stroke | 388 (5.2) | 210 (5.3) | 123 (5.8) | 0.556 |
| Serious illness in the past 2 years | 1181 (15.7) | 689 (17.1) | 385 (17.7) | **0.034** |
| Hearing problem | 736 (9.8) | 1072 (26.5) | 426 (19.6) | **<0.001** |
| Visual impairment | 813 (10.8) | 962 (23.8) | 418 (19.3) | **<0.001** |
| **Adverse health outcomes** |  |  |  |  |
| Cognitive impairment | 819 (10.9) | 1309 (32.4) | 462 (21.4) | **<0.001** |
| Functional limitation | 2406 (32.0) | 2681 (66.3) | 1091 (50.2) | **<0.001** |
| Frailty | 939 (12.5) | 1658 (41.0) | 617 (28.4) | **<0.001** |

*Note.* Data presented as n (%) or mean (SD). SDW, Single/Separated/Divorced/Widowed.

**Table S2. Modification of the Effect of Loneliness on Cognitive Impairment by Living Arrangement According to Age and Gender**

| **Cognitive Impairment** | | ***<80 years*** | |  | ***≥80 years*** | |  | ***Male*** | |  | ***Female*** | |
| --- | --- | --- | --- | --- | --- | --- | --- | --- | --- | --- | --- | --- |
|  |  | **OR (95% CI)** | ***P*** |  | **OR (95% CI)** | ***P*** |  | **OR (95% CI)** | ***P*** |  | **OR (95% CI)** | ***P*** |
| **Cross-sectional Analyses ^a^** | |  |  |  |  |  |  |  |  |  |  |  |
| **Loneliness** | |  |  |  |  |  |  |  |  |  |  |  |
| NFL | | 1.00 |  |  | 1.00 |  |  | 1.00 |  |  | 1.00 |  |
| FL | | 1.26 (0.79-2.01) | 0.335 |  | 1.24 (1.08-1.41) | **0.002** |  | 1.23 (0.99-1.53) | 0.057 |  | 1.21 (1.03-1.42) | **0.022** |
| **Living arrangements** | **Loneliness** |  |  |  |  |  |  |  |  |  |  |  |
| NLA | NFL | 1.00 |  |  | 1.00 |  |  | 1.00 |  |  | 1.00 |  |
| NLA | FL | 1.39 (0.81-2.40) | 0.230 |  | 1.34 (1.16-1.55) | **<0.001** |  | 1.36 (1.07-1.72) | **0.012** |  | 1.32 (1.10-1.57) | **0.002** |
| LA | NFL | 1.00 |  |  | 1.00 |  |  | 1.00 |  |  | 1.00 |  |
| LA | FL | 0.58 (0.18-1.89) | 0.363 |  | 0.80 (0.57-1.11) | 0.179 |  | 0.64 (0.37-1.11) | 0.114 |  | 0.77 (0.51-1.16) | 0.215 |
| **Longitudinal Analyses ^b^** | |  |  |  |  |  |  |  |  |  |  |  |
| **Loneliness** | |  |  |  |  |  |  |  |  |  |  |  |
| NFL | | 1.00 |  |  | 1.00 |  |  | 1.00 |  |  | 1.00 |  |
| FL | | 0.86 (0.51-1.46) | 0.583 |  | 1.02 (0.79-1.33) | 0.857 |  | 1.40 (0.96-2.06) | 0.083 |  | 0.83 (0.62-1.11) | 0.218 |
| **Living arrangements** | **Loneliness** |  |  |  |  |  |  |  |  |  |  |  |
| NLA | NFL | 1.00 |  |  | 1.00 |  |  | 1.00 |  |  | 1.00 |  |
| NLA | FL | 0.93 (0.51-1.71) | 0.820 |  | 1.03 (0.77-1.39) | 0.818 |  | 1.59 (1.03-2.44) | **0.036** |  | 0.84 (0.60-1.17) | 0.296 |
| LA | NFL | 1.00 |  |  | 1.00 |  |  | 1.00 |  |  | 1.00 |  |
| LA | FL | - | - |  | 1.09 (0.52-2.27) | 0.817 |  | - | - |  | 0.70 (0.31-1.59) | 0.395 |

*Note.* NLA, not living alone; LA, living alone; NFL, not feeling lonely; FL, feeling lonely. Same adjustment as Table 3.

**^a^** Measures of effect modifications on multiplicative scale: (1) <80 years: OR (95% CI) = 0.65 (0.23-1.90), *P*=0.434; (2) ≥80 years: OR (95% CI) = 0.62 (0.44-0.87), *P*=0.006; (3) Male: OR (95% CI) = 0.61 (0.36-1.05), *P*=0.076; (4) Female: OR (95% CI) = 0.61 (0.40-0.92), *P*=0.018.

**^b^** Measures of effect modifications on multiplicative scale: (1) <80 years: OR (95% CI) = 0.55 (0.17-1.77), *P*=0.317; (2) ≥80 years: OR (95% CI) = 0.94 (0.51-1.74), *P*=0.850; (3) Male: OR (95% CI) = 0.81 (0.31-2.10), *P*=0.666; (4) Female: OR (95% CI) = 0.96 (0.50-1.87), *P*=0.909.

**Table S3. Modification of the Effect of Loneliness on Functional Limitation by Living Arrangement According to Age and Gender**

| **Functional limitation** | | ***<80 years*** | |  | ***≥80 years*** | |  | ***Male*** | |  | ***Female*** | |
| --- | --- | --- | --- | --- | --- | --- | --- | --- | --- | --- | --- | --- |
|  |  | **OR (95% CI)** | ***P*** |  | **OR (95% CI)** | ***P*** |  | **OR (95% CI)** | ***P*** |  | **OR (95% CI)** | ***P*** |
| **Cross-sectional Analyses** | |  |  |  |  |  |  |  |  |  |  |  |
| **Loneliness** | |  |  |  |  |  |  |  |  |  |  |  |
| NFL | | 1.00 |  |  | 1.00 |  |  | 1.00 |  |  | 1.00 |  |
| FL | | 1.09 (0.84-1.43) | 0.518 |  | 1.06 (0.94-1.19) | 0.346 |  | 1.06 (0.89-1.25) | 0.524 |  | 1.07 (0.93-1.23) | 0.364 |
| **Living arrangements** | **Loneliness** |  |  |  |  |  |  |  |  |  |  |  |
| NLA | NFL | 1.00 |  |  | 1.00 |  |  | 1.00 |  |  | 1.00 |  |
| NLA | FL | 1.20 (0.89-1.61) | 0.230 |  | 1.06 (0.92-1.21) | 0.428 |  | 1.04 (0.86-1.26) | 0.696 |  | 1.12 (0.95-1.31) | 0.183 |
| LA | NFL | 1.00 |  |  | 1.00 |  |  | 1.00 |  |  | 1.00 |  |
| LA | FL | 0.71 (0.35-1.43) | 0.336 |  | 1.07 (0.83-1.38) | 0.612 |  | 1.11 (0.74-1.65) | 0.621 |  | 0.91 (0.68-1.23) | 0.552 |
| **Longitudinal Analyses** | |  |  |  |  |  |  |  |  |  |  |  |
| **Loneliness** | |  |  |  |  |  |  |  |  |  |  |  |
| NFL | | 1.00 |  |  | 1.00 |  |  | 1.00 |  |  | 1.00 |  |
| FL | | 1.07 (0.79-1.45) | 0.676 |  | 1.15 (0.88-1.48) | 0.305 |  | 1.13 (0.84-1.52) | 0.431 |  | 1.02 (0.78-1.33) | 0.873 |
| **Living arrangements** | **Loneliness** |  |  |  |  |  |  |  |  |  |  |  |
| NLA | NFL | 1.00 |  |  | 1.00 |  |  | 1.00 |  |  | 1.00 |  |
| NLA | FL | 1.06 (0.74-1.51) | 0.762 |  | 1.13 (0.83-1.53) | 0.446 |  | 1.04 (0.74-1.47) | 0.818 |  | 1.04 (0.76-1.43) | 0.795 |
| LA | NFL | 1.00 |  |  | 1.00 |  |  | 1.00 |  |  | 1.00 |  |
| LA | FL | 0.93 (0.40-2.12) | 0.856 |  | 1.39 (0.76-2.53) | 0.284 |  | 1.56 (0.72-3.37) | 0.258 |  | 0.96 (0.52-1.75) | 0.888 |

*Note.* NLA, not living alone; LA, living alone; NFL, not feeling lonely; FL, feeling lonely. Same adjustment as Table 4.

**^a^** Measures of effect modifications on multiplicative scale: (1) <80 years: OR (95% CI) = 0.56 (0.29-1.11), *P*=0.099; (2) ≥80 years: OR (95% CI) = 1.00 (0.75-1.31), *P*=0.974; (3) Male: OR (95% CI) = 1.00 (0.66-1.52), *P*=0.993; (4) Female: OR (95% CI) = 0.81 (0.59-1.13), *P*=0.220.

**^b^** Measures of effect modifications on multiplicative scale: (1) <80 years: OR (95% CI) = 0.97 (0.49-1.93), *P*=0.923; (2) ≥80 years: OR (95% CI) = 1.09 (0.62-1.92), *P*=0.762; (3) Male: OR (95% CI) = 1.23 (0.61-2.46), *P*=0.564; (4) Female: OR (95% CI) = 0.89 (0.50-1.58), *P*=0.690.

**Table S4. Modification of the Effect of Loneliness on Frailty by Living Arrangement According to Age and Gender**

| **Frailty** | | ***<80 years*** | |  | ***≥80 years*** | |  | ***Male*** | |  | ***Female*** | |
| --- | --- | --- | --- | --- | --- | --- | --- | --- | --- | --- | --- | --- |
|  |  | **OR (95% CI)** | ***P*** |  | **OR (95% CI)** | ***P*** |  | **OR (95% CI)** | ***P*** |  | **OR (95% CI)** | ***P*** |
| **Cross-sectional Analyses** | |  |  |  |  |  |  |  |  |  |  |  |
| **Loneliness** | |  |  |  |  |  |  |  |  |  |  |  |
| NFL | | 1.00 |  |  | 1.00 |  |  | 1.00 |  |  | 1.00 |  |
| FL | | 1.87 (1.34-2.63) | **<0.001** |  | 1.36 (1.20-1.53) | **<0.001** |  | 1.46 (1.21-1.76) | **<0.001** |  | 1.38 (1.20-1.58) | **<0.001** |
| **Living arrangements** | **Loneliness** |  |  |  |  |  |  |  |  |  |  |  |
| NLA | NFL | 1.00 |  |  | 1.00 |  |  | 1.00 |  |  | 1.00 |  |
| NLA | FL | 1.87 (1.29-2.69) | **0.001** |  | 1.36 (1.20-1.55) | **<0.001** |  | 1.53 (1.25-1.88) | **<0.001** |  | 1.33 (1.15-1.55) | **<0.001** |
| LA | NFL | 1.00 |  |  | 1.00 |  |  | 1.00 |  |  | 1.00 |  |
| LA | FL | 2.76 (0.96-7.94) | 0.060 |  | 1.35 (0.99-1.83) | 0.057 |  | 1.04 (0.63-1.72) | 0.874 |  | 1.66 (1.15-2.39) | **0.006** |
| **Longitudinal Analyses** | |  |  |  |  |  |  |  |  |  |  |  |
| **Loneliness** | |  |  |  |  |  |  |  |  |  |  |  |
| NFL | | 1.00 |  |  | 1.00 |  |  | 1.00 |  |  | 1.00 |  |
| FL | | 1.26 (0.84-1.89) | 0.262 |  | 1.18 (0.95-1.48) | 0.142 |  | 1.10 (0.79-1.54) | 0.557 |  | 1.29 (1.01-1.65) | **0.042** |
| **Living arrangements** | **Loneliness** |  |  |  |  |  |  |  |  |  |  |  |
| NLA | NFL | 1.00 |  |  | 1.00 |  |  | 1.00 |  |  | 1.00 |  |
| NLA | FL | 1.35 (0.84-2.16) | 0.212 |  | 1.16 (0.90-1.50) | 0.248 |  | 1.19 (0.82-1.73) | 0.361 |  | 1.25 (0.94-1.66) | 0.130 |
| LA | NFL | 1.00 |  |  | 1.00 |  |  | 1.00 |  |  | 1.00 |  |
| LA | FL | 1.09 (0.40-3.00) | 0.867 |  | 1.03 (0.63-1.68) | 0.908 |  | 0.76 (0.33-1.74) | 0.512 |  | 1.27 (0.77-2.10) | 0.341 |

*Note.* NLA, not living alone; LA, living alone; NFL, not feeling lonely; FL, feeling lonely. Same adjustment as Table 5.

**^a^** Measures of effect modifications on multiplicative scale: (1) <80 years: OR (95% CI) = 0.99 (0.39-2.51), *P*=0.976; (2) ≥80 years: OR (95% CI) = 0.98 (0.71-1.35), *P*=0.885; (3) Male: OR (95% CI) = 0.65 (0.39-1.09), *P*=0.104; (4) Female: OR (95% CI) = 1.27 (0.86-1.86), *P*=0.223.

**^b^** Measures of effect modifications on multiplicative scale: (1) <80 years: OR (95% CI) = 0.79 (0.32-1.96), *P*=0.610; (2) ≥80 years: OR (95% CI) = 1.01 (0.59-1.71), *P*=0.976; (3) Male: OR (95% CI) = 0.70 (0.31-1.57), *P*=0.382; (4) Female: OR (95% CI) = 1.07 (0.61-1.88), *P*=0.803.

**Table S5. Modification of the Effect of Loneliness on 3-year Mortality by Living Arrangement According to Age and Gender**

| **Mortality** | | ***<80 years*** | |  | ***≥80 years*** | |  | ***Male*** | |  | ***Female*** | |
| --- | --- | --- | --- | --- | --- | --- | --- | --- | --- | --- | --- | --- |
|  |  | **HR (95% CI)** | ***P*** |  | **HR (95% CI)** | ***P*** |  | **HR (95% CI)** | ***P*** |  | **HR (95% CI)** | ***P*** |
| **Cross-sectional Analyses** | |  |  |  |  |  |  |  |  |  |  |  |
| **Loneliness** | |  |  |  |  |  |  |  |  |  |  |  |
| NFL | | 1.00 |  |  | 1.00 |  |  | 1.00 |  |  | 1.00 |  |
| FL | | 1.50 (1.16-1.93) | **0.002** |  | 1.07 (0.99-1.16) | 0.078 |  | 1.07 (0.95-1.21) | 0.242 |  | 1.13 (1.02-1.25) | **0.015** |
| **Living arrangements** | **Loneliness** |  |  |  |  |  |  |  |  |  |  |  |
| NLA | NFL | 1.00 |  |  | 1.00 |  |  | 1.00 |  |  | 1.00 |  |
| NLA | FL | 1.55 (1.17-2.07) | **0.002** |  | 1.11 (1.02-1.21) | **0.016** |  | 1.12 (0.98-1.27) | 0.096 |  | 1.16 (1.04-1.30) | **0.006** |
| LA | NFL | 1.00 |  |  | 1.00 |  |  | 1.00 |  |  | 1.00 |  |
| LA | FL | 1.32 (0.68-2.56) | 0.417 |  | 0.88 (0.72-1.07) | 0.208 |  | 0.85 (0.65-1.13) | 0.268 |  | 0.93 (0.71-1.23) | 0.616 |

*Note.* NLA, not living alone; LA, living alone; NFL, not feeling lonely; FL, feeling lonely. Same adjustment as Table 6.

Measures of effect modifications on multiplicative scale: (1) <80 years: OR (95% CI) = 0.80 (0.38-1.66), *P*=0.543; (2) ≥80 years: OR (95% CI) = 0.72 (0.54-0.96), *P*=0.025; (3) Male: OR (95% CI) = 0.76 (0.51-1.14), *P*=0.184; (4) Female: OR (95% CI) = 0.72 (0.50-1.03), *P*=0.070.
