# Supplementary figures and images for: Living arrangement modifies the associations of loneliness with adverse health outcomes in older adults: evidence from the CLHLS
Source: BMC Geriatr. 2022 Jan 17;22:59. doi: 10.1186/s12877-021-02742-5 (PMC8764854; doi:10.1186/s12877-021-02742-5)

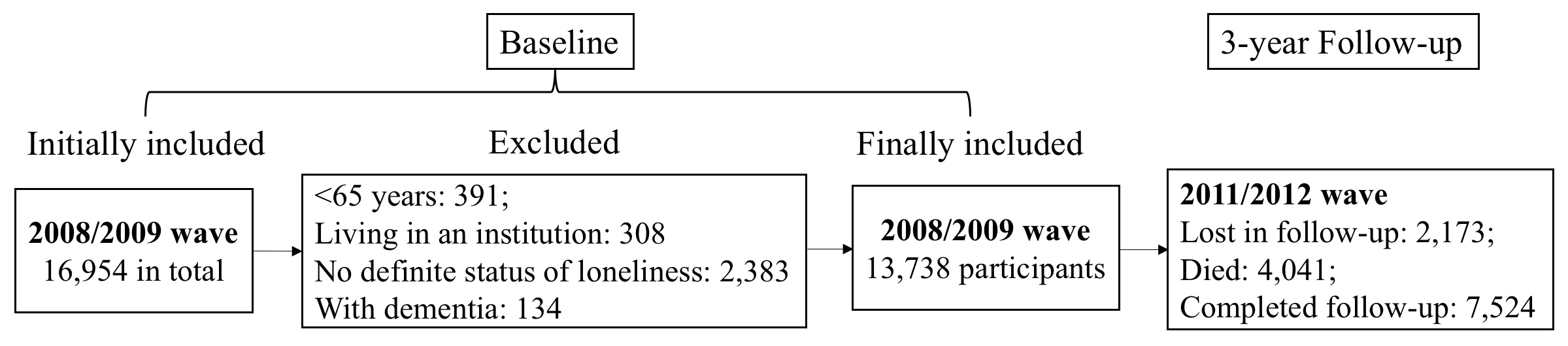

Supplement: Supplementary file 2 — Additional file 2. [file 12877_2021_2742_MOESM2_ESM.jpg]
